# Real-World Evidence From a Digital Health Treatment Program for Female Urinary Incontinence: Observational Study of Outcomes Following User-Centered Product Design

**DOI:** 10.2196/58551

**Published:** 2024-06-27

**Authors:** Evelyn Hall, Laura Keyser, Jessica McKinney, Samantha Pulliam, Milena Weinstein

**Affiliations:** 1 Tufts University Medical Center Tufts University School of Medicine Boston, MA United States; 2 Department of Physical Therapy and Rehabilitation Science University of California, San Francisco San Francisco, CA United States; 3 Axena Health, Inc. Auburndale, MA United States; 4 College of Health and Human Services Andrews University Berrien Springs, MI United States; 5 Massachusetts General Hospital Harvard Medical School Boston, MA United States

**Keywords:** urinary incontinence, digital health, pelvic floor muscle training, real-world, evidence, user-centered design, mobile phone

## Abstract

**Background:**

Urinary incontinence (UI) affects millions of women with substantial health and quality-of-life impacts. Supervised pelvic floor muscle training (PFMT) is the recommended first-line treatment. However, multiple individual and institutional barriers impede women’s access to skilled care. Evidence suggests that digital health solutions are acceptable and may be effective in delivering first-line incontinence treatment, although these technologies have not yet been leveraged at scale.

**Objective:**

The primary objective is to describe the effectiveness and safety of a prescribed digital health treatment program to guide PFMT for UI treatment among real-world users. The secondary objectives are to evaluate patient engagement following an updated user platform and identify the factors predictive of success.

**Methods:**

This retrospective cohort study of women who initiated device use between January 1, 2022, and June 30, 2023, included users aged ≥18 years old with a diagnosis of stress, urgency, or mixed incontinence or a score of >33.3 points on the Urogenital Distress Inventory Short Form (UDI-6). Users are prescribed a 2.5-minute, twice-daily, training program guided by an intravaginal, motion-based device that pairs with a smartphone app. Data collected by the device or app include patient-reported demographics and outcomes, adherence to the twice-daily regimen, and pelvic floor muscle performance parameters, including angle change and hold time. Symptom improvement was assessed by the UDI-6 score change from baseline to the most recent score using paired 2-tailed *t* tests. Factors associated with meeting the UDI-6 minimum clinically important difference were evaluated by regression analysis.

**Results:**

Of 1419 users, 947 met inclusion criteria and provided data for analysis. The mean baseline UDI-6 score was 46.8 (SD 19.3), and the mean UDI-6 score change was 11.3 (SD 19.9; *P*<.001). Improvement was reported by 74% (697/947) and was similar across age, BMI, and incontinence subtype. Mean adherence was 89% (mean 12.5, SD 2.1 of 14 possible weekly uses) over 12 weeks. Those who used the device ≥10 times per week were more likely to achieve symptom improvement. In multivariate logistic regression analysis, baseline incontinence symptom severity and maximum angle change during pelvic floor muscle contraction were significantly associated with meeting the UDI-6 minimum clinically important difference. Age, BMI, and UI subtype were not associated.

**Conclusions:**

This study provides real-world evidence to support the effectiveness and safety of a prescribed digital health treatment program for female UI. A digital PFMT program completed with visual guidance from a motion-based device yields significant results when executed ≥10 times per week over a period of 12 weeks. The program demonstrates high user engagement, with 92.9% (880/947) of users adhering to the prescribed training regimen. First-line incontinence treatment, when implemented using this digital program, leads to statistically and clinically substantial symptom improvements across age and BMI categories and incontinence subtypes.

## Introduction

Digital health is an umbrella term that refers to the use of information and communications technologies in medicine and other health professions to manage illnesses and health risks and to promote wellness [1]. Examples include telemedicine, remote sensors and monitors, digital health records and data analytics, and predictive modeling. Digital health technologies afford the opportunity both to deliver personalized medicine and to scale up effective interventions to meet population health needs, particularly in light of gross health care worker shortages [2]. A key feature of digital health technologies is the ability to collect robust real-world data, which may be used to drive improvements in product design, promote uptake and adherence, and demonstrate population effectiveness in support of regulatory and reimbursement efforts. Historically overlooked and underresourced, there is a potential for women’s health to be revolutionized by digital health by addressing gendered barriers to care, accelerating the development of diagnostics and therapeutics, and conveying impacts across the life course in reproductive health, maternal health, sexual health, and menopause care [3].

Pelvic floor disorders and urinary incontinence (UI), in particular, disproportionately affect women. Prevalence estimates indicate that over 60% of women in the United States experience UI, with over 28 million women reporting bothersome symptoms [4]. Robust evidence supports pelvic floor muscle training (PFMT) as a first-line intervention for the 3 predominant UI subtypes, which are stress, urgency, and mixed UI [5]. Data suggest that approximately 67% of women experience symptom improvement or resolution with PFMT [6]. Despite high UI prevalence and strong evidence to support behavioral interventions for UI, most women are untreated or undertreated for these conditions [7-9]. Health care workforce shortages, including a lack of physical therapists skilled in pelvic floor disorders care, contribute to these low levels of UI treatment [8]. Evidence suggests that digital health solutions are acceptable and may be effective in delivering first-line UI treatment. However, to date, these technologies have not been leveraged at scale [10-13].

The primary objective of this study is to describe the effectiveness and safety of a prescribed digital health treatment program for UI using a motion-based device to guide PFMT for the first-line treatment of stress, urgency, and mixed UI among a cohort of real-world users. The secondary objectives are to evaluate patient engagement following an updated user platform and to identify factors predictive of treatment success.

## Methods

### Overview

This is a retrospective cohort study of real-world users of a prescribed digital health treatment program for UI using a motion-based device who initiated treatment between January 1, 2022, and June 30, 2023. All users were included who were aged ≥18 years with a UI diagnosis (*International Classification of Diseases, Tenth Revision* [*ICD-10*] N39.3, N39.41, N32.81, and N39.46) or, in the absence of any diagnosis code, a baseline Urogenital Distress Inventory Short Form (UDI-6) score indicative of symptomatic UI (score >33.3) [14]. Users who provided baseline and at least one follow-up UDI-6 score at or after 4 weeks were included. Users with a non-UI diagnosis were excluded.

The Leva Pelvic Health System (Axena Health Inc) is a prescription medical device commercially available in the United States that combines hardware (an intravaginal sensor) and software (a smartphone app) to guide PFMT. The hardware component detects the movement produced during pelvic floor muscle (PFM) contraction and relaxation. This information is reflected in real time through software that gives the user visual feedback about their PFM performance. Training sessions are 2.5 minutes each, and users are instructed to complete these twice daily for a maximum of 14 weekly sessions over a period of 12 weeks. During training, users are directed to lift and squeeze the PFM for a period of 15 seconds, followed by a 15-second guided relaxation period. PFMT data captured by the software include adherence to the twice-daily training program, the maximum angle change associated with each PFM contraction, and the average hold time of the PFM contraction maintained during the training program (maximum 15 seconds).

In addition to PFMT sessions, the product software facilitates user engagement and adherence in the form of written and video educational content, motivational messaging, symptom tracking through validated questionnaires, and PFM performance monitoring. Users have the opportunity to provide demographic and health information and are prompted to complete the UDI-6 at baseline, 4, 8, and 12 weeks. The UDI-6 is a validated survey that assesses the presence and degree of bother of urinary symptoms and is sensitive to symptom change over time. Scores range from 0 to 100 points, with a score reduction indicating symptom improvement [15]. At 12 weeks, all users are prompted to complete the Patient Global Impression of Improvement (PGI-I), a single-question survey delivered through SMS text message. The PGI-I has been validated for the assessment of UI symptom change [16]. Users are also assigned a designated coach, who, for those who opt-in, provides technical and motivational support for a period of 12 weeks. The program is designed to be completed over a 12-week period, though some users continue to use the device and complete the UDI-6 survey beyond 12 weeks. Adverse events were reported through regular SMS text messages or phone calls with coaches.

Participants included commercial users who were identified by screening the user database to identify the appropriate *ICD-10* code or UDI-6 score threshold. All data were deidentified before analysis. Data collected included patient-reported age, BMI, race, ethnicity, health information, baseline and follow-up UDI-6 scores, and adverse events. Race and ethnicity were collected to understand if those who used the device were representative of the population in the United States. Device-reported data included adherence (number of uses per week) and PFM performance parameters (angle change during PFM contraction and PFM contraction hold time).

### Outcomes Analysis

The primary outcome of effectiveness was evaluated for participants who provided baseline and at least one follow-up UDI-6 survey completed at or after 4 weeks of use. Demographics and other baseline data were summarized and compared with individuals who did not provide follow-up UDI-6 surveys to assess differences between user groups using student 2-tailed *t* tests. Effectiveness was measured in three ways, that are (1) the proportion of users who met the minimum clinically important difference (MCID) on the UDI-6; (2) the proportion of users who met the Patient Acceptable Symptom State (PASS) on the UDI-6; and (3) the proportion of users who indicated “very much better,” “much better,” or “a little better” on the PGI-I. Symptom improvement was assessed by the UDI-6 score change from baseline to the most recently reported score. Paired *t* tests were used to assess the UDI-6 score change from baseline to follow-up. Subgroup analysis by age (18-44, 45-64, and ≥65 years), BMI (underweight or <18.5 kg/m^2^, healthy weight or 18.5-25 kg/m^2^, overweight or >25-30 kg/m^2^, and obese or >30 kg/m^2^), and UI subtype (stress, mixed, urgency, and unspecified) was completed to determine differences across categories. The proportion of participants reaching the MCID was calculated by converting the UDI-6 score to a UDI-6 Long Form score and then evaluating the number reaching a change in score of 11 points or more [17]. The PASS represents the point at which a patient considers themselves well and is thus unlikely to seek additional treatment for their health condition. PASS was evaluated by counting the number of participants who reached the cutoff score identified for the UDI-6 of 37.5 [18].

Adherence to the prescribed PFMT regimen was averaged over a 12-week period and categorized into 3 groups, such as 0-4 uses per week, 5-9 uses per week, and ≥10 uses per week, with a maximum of 14 weekly uses. Non-responses were considered missing data and excluded from analysis. Multimedia Appendix 1 provides further analysis of adherence, in which non-responses were considered non-use (0 uses). A linear mixed model was used to evaluate the impact of maximum angle change with PFM contraction on the attainment of UDI-6 MCID. A logistic regression analysis was performed to assess factors associated with meeting the UDI-6 MCID. Covariates included age, BMI, baseline UDI-6 score, adherence category, UI subtype, PFM angle change, and PFM contraction hold time change. Data analysis was completed using SPSS (version 28.0.0.1; IBM Corp) and R (version 4.3.2; R Core Team).

### Ethical Considerations

This study was exempted from review under 45 CFR § 46.104(d)(4) by the Western-Copernicus Group Institutional Review Board. Before device use, all users provide consent for the capture and storage of their personal and device-related data in a Health Insurance Portability and Accountability Act (HIPAA)–compliant manner. Participants were assigned a 32-character alphanumeric identifier. These deidentified data were extracted from the commercial user database, transmitted securely using an encrypted connection, and stored in an encrypted and password-protected research database. No compensation was provided to participants, as this was a retrospective review of real-world users.

## Results

**Figure 1:**
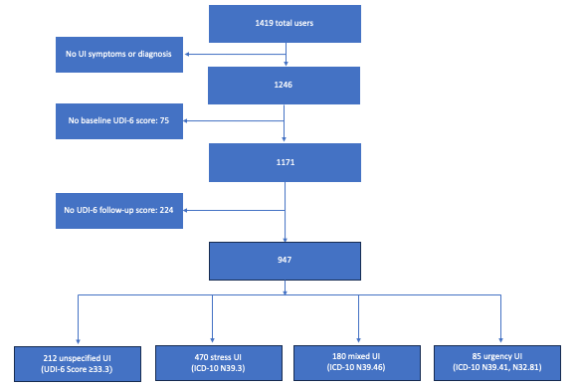
Flow diagram of real-world users of the digital health treatment program for urinary incontinence (UI) using a motion-based device included in this analysis. *ICD-10: International Classification of Diseases, Tenth Revision*; UDI-6: Urogenital Distress Inventory-6 Short Form.

Of 1419 users, 947 met inclusion criteria and provided data for analysis (Figure 1). Demographic data and clinical characteristics are summarized in Table 1. Of the 947 included participants, the mean age was 51 years, and the mean BMI was 28.3 kg/m^2^. The included participants were compared with 224 users who provided baseline UDI-6 data only. Those with complete data and included in the outcomes analysis were older, had a slightly lower BMI, and were more adherent to training (Table 1).

The mean baseline UDI-6 score was 46.8 (SD 19.3) points, and the mean UDI-6 score change was 11.3 (SD 19.9; *P*<.001). The mean time to follow-up (time between baseline and most recent UDI-6 follow-up) was 14.6 (SD 8.7, median 12, IQR 8-26) weeks. Improvement in UDI-6 scores was observed across users, regardless of age, BMI, and UI subtype, and symptom improvement was similar across these categories (Table 2). Evaluation of treatment effectiveness as measured by UDI-6 demonstrated that 58.4% (553/947) met the MCID and 60.6% (574/947) met the cutoff for PASS. About 73.6% of users (697/947) experienced improvement by meeting at least one of these measures. Of 651 PGI-I responses, 77% (501/651) reported improvement. Worsening symptoms were reported by 2.7% (18/651).

Overall mean adherence for the group who provided baseline and follow-up UDI-6 scores was 89% (mean 12.5, SD 2.1 of 14 possible uses) over 12 weeks. Comparison across adherence categories demonstrated that only those in the ≥10 uses per week group reported significant UDI-6 score improvement (*P*<.001). This group was more likely to reach the MCID compared with those who were in the 0-4 or 5-9 uses per week groups (Table 3).

**Table 1:** A summary and comparison of user demographics and baseline characteristics for those who provided follow-up urinary incontinence outcomes data and those who provided baseline data only.

Demographics			All participants (n=1246)		Baseline UDI-6^a^ scores only (n=224)		Baseline and follow-up UDI-6 scores (n=947)		*P* value
Age (years), mean (SD)			50.3 (11.8)		47.6 (12.1)		51 (11.6)		<.001^b^
**Race, n (%)**									.96
	Asian	35 (2.8)		5 (1.7)		30 (3.2)			
	Black	74 (5.9)		13 (4.4)		61 (6.4)			
	White	891 (71.5)		168 (56.2)		723 (76.4)			
	American Indian or Alaskan Native	5 (0.4)		0 (0)		5 (0.5)			
	Native Hawaiian or Pacific Islander	3 (0.2)		0 (0)		3 (0.3)			
	Other	62 (5)		9 (3)		53 (5.6)			
	Unknown	17 (1.4)		3 (1)		14 (1.5)			
	Not reported	159 (12.8)		101 (33.8)		58 (6.1)			
**Ethnicity, n (%)**									.26
	Hispanic or Latino	72 (5.8)		16 (5.4)		56 (5.9)			
	Not Hispanic or Latino	835 (67)		142 (47.5)		693 (73.2)			
	Not reported	339 (27.2)		141 (47.2)		198 (21)			
BMI (kg/m^2^), mean (SD)			28.5 (6.6)		29.7 (7.4)		28.3 (6.4)		.01^b^
Adherence (weekly uses over 12 weeks), median (IQR)			12.7 (11.0-13.8)		11.7 (9.3-13.5)		12.9 (11.4-13.9)		**<**.001^b^
**Childbirth, n (%)**									.96
	Has given birth	635 (51)		64 (21.4)		571 (60.3)			
	Never given birth	67 (5.4)		5 (1.7)		62 (6.6)			
	Not reported	542 (43.5)		229 (76.6)		313 (33.1)			
Baseline UDI-6 score, mean (SD)			47 (19.8)		47.9 (21.8)		46.8 (19.3)		.49
Baseline hold time (seconds), mean (SD)			6.4 (3.5)		6.2 (3.4)		6.4 (3.5)		.39
Baseline angle change (degrees), mean (SD)			10.4 (5.9)		10.1 (5.3)		10.5 (6.1)		.31

^a^UDI-6: Urogenital Distress Inventory-6 Short Form.

^b^Values are significant.

**Table 2:** Urinary incontinence (UI) outcomes from a single cohort of real-world users: Urogenital Distress Inventory-6 (UDI-6) improvement from baseline to follow-up by age, BMI, and UI subtype.

Subtypes		Participants, n	Baseline UDI-6, mean (SD)	Last reported UDI-6, mean (SD)	UDI-6 mean difference, mean (SD)	*P* value^a^		Positive clinical improvement, n (%)		*P* value^b^		
**Age (years)**							<.001				.75	
	18-44	164	46.7 (18.8)	35.7 (24.5)	11.03 (22.2)			119 (72.6)				
	45-65	636	46.7 (19.6)	35.5 (21.2)	11.20 (19.6)			472 (74.2)				
	≥65	124	47 (19.1)	34.3 (21.3)	12.6 (18.5)			89 (71.8)				
**BMI (kg/m^2^)**												.07
	Underweight: <18.5 kg/m^2^	9	43.5 (17.2)	44 (33.9)	–0.46 (26.8)	.96		4 (44.4)				
	Healthy weight: 18.5-25 kg/m^2^	316	41.7 (18.5)	31.7 (20.5)	9.91 (20)	<.001		233 (73.7)				
	Overweight: >25-30 kg/m^2^	276	48 (18)	35.1 (22.2)	12.9 (20)	<.001		205 (74.3)				
	Obese: >30 kg/m^2^	227	51 (20.1)	38.7 (21.9)	12.4 (19.7)	<.001		227 (74.2)				
**UI subtype**							<.001				.42	
	Stress UI	456	42.4 (19.4)	32 (21.3)	10.4 (19.6)			370 (87.7)				
	Mixed UI	176	50.4 (20.6)	38.7 (23.2)	11.7 (20.6)			131 (72.8)				
	Urgency UI	85	46.7 (19)	36.7 (20.9)	9.9 (19.1)			60 (70.6)				
	Unspecified UI	207	53.3 (15.5)	40 (20.6)	13.3 (20.1)			136 (64.2)				

^a^Paired *t* test results.

^b^Chi-square test results.

**Table 3:** Urinary incontinence outcomes from a single cohort of real-world users: Urogenital Distress Inventory-6 Short Form (UDI-6) changes from baseline to follow-up by adherence category. ANOVA <.001.

Adherence category	Participants, n	Baseline UDI-6, mean (SD)	UDI-6 at last follow-up, mean (SD)	Mean difference (SD)	*P* value
0-4 uses per week	19	44.1 (16.7)	46.1 (21)	–2 (17.1)	.62
5-9 uses per week	48	48.3 (22.1)	44.7 (21.3)	3.6 (17.7)	.17
≥10 uses per week	880	46.8 (19.2)	34.8 (21.6)	12 (19.9)	<.001

**Figure 2:**
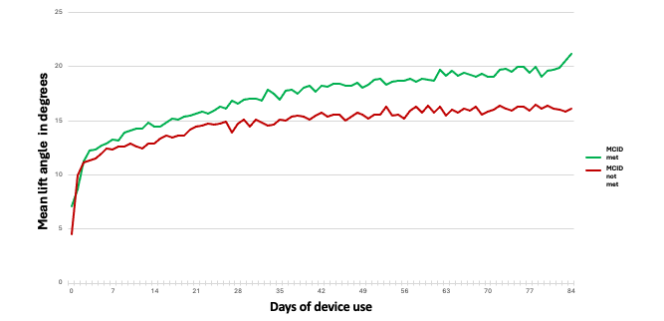
Maximum angle change with pelvic floor muscle (PFM) contraction depicted over time for individuals who used the device 10+ times per week over 12-weeks who met and did not meet the minimum clinically important difference (MCID) on the Urogenital Distress Inventory-6 Short Form (UDI-6). Individuals who met the MCID demonstrate greater angle change during PFM contraction compared with those who do not, and this difference is evident early in their training and persists over time.

Overall, PFM performance parameters improved from baseline to follow-up. Maximum angle change during PFM contraction increased from mean 10.5 (SD 6.1) degrees at baseline to mean 18.4 (SD 8.7) degrees at the 12-week period (*P*<.001). Mean PFM contraction hold time increased from 6.4 (SD 3.5) seconds at baseline to 10.2 (SD 3.2) seconds at the 12-week period (*P*<.001). Among participants who used the device 10 or more times weekly, those who reached the UDI-6 MCID demonstrated significantly greater mean angle change and hold time compared with those who did not (Figure 2). In multivariate logistic regression analysis, UI symptom severity as reported on the baseline UDI-6 score and maximum angle change during PFM contraction were significantly associated with meeting the MCID (Table 4). Age, BMI, and UI subtype were not associated.

Adverse events included vaginal irritation (32/1419, 2.3%), back pain (27/1419, 1.9%), yeast infection (10/1419, 0.7%), and urinary tract infection (9/1419, 0.6%). No serious adverse events were reported.

**Table 4:** Urinary incontinence (UI) outcomes from a single cohort of real-world users: factors associated with meeting the Urogenital Distress Inventory-6 Short Form (UDI-6) minimum clinically important difference.

Covariates		Adjusted odds ratio (95% CIs)	*P* value
Age		1.0098 (0.9922-1.0277)	.28
BMI		1.0175 (0.9825-1.0538)	.33
Baseline UDI-6 score		1.0426 (1.0303-1.0552)	<.001^a^
Adherence (weekly uses over the 12-week program)		0.9072 (0.8041-1.0235)	.11
Baseline maximum angle change with PFM^b^ contraction		0.9951 (0.9537-1.0382)	.82
Follow-up maximum angle change with PFM contraction		1.0359 (1.0151-1.0570)	.001^a^
**UI subtypes**			
	Urgency UI	Reference (—^c^)	—
	Mixed UI	0.7751 (0.3399-1.7672)	.55
	Stress UI	1.1824 (0.5518-2.5338)	.67
	Unspecified UI	0.6366 (0.2818-1.4381)	.28

^a^Values are significant.

^b^PFM: pelvic floor muscle.

^c^Not applicable.

## Discussion

### Overview

This study adds real-world evidence to support a prescribed digital health treatment program for stress, urgency, and mixed UI using a motion-based device. UI symptom improvement, as measured by achieving the UDI-6 MCID, was observed in 58.4% (553/947) of users. About 60.6% (574/947) of users achieved the UDI-6 threshold for PASS, and 73.6% (697/947) achieved either MCID or PASS, regardless of age, BMI, childbirth history, or UI subtype. Participants who adhered to a recommended regimen by completing at least 10 PFMT sessions per week were more likely to achieve MCID compared with those who did not. In addition, the baseline UDI-6 score and device-reported maximum angle change with PFM contraction were associated with achieving the UDI-6 MCID. Device use yields a high safety profile, with very few reported adverse events and no serious adverse events.

This study confirms previous findings describing real-world evidence using the same motion-based device, though the larger sample of users enabled a more detailed evaluation of the findings [13]. In contrast with the previous study, adherence significantly influenced symptom improvement in this study. Historically, it has been challenging to measure adherence to PFMT, and to date, there is no consensus regarding the ideal frequency and duration of PFMT required to achieve substantial UI symptom improvement. However, it is known that increased frequency, intensity, and supervision are more effective [5]. This study demonstrates that a digital PFMT program completed with visual guidance from a motion-based device yields substantial results when executed ≥10 times per week over a period of 12 weeks. Furthermore, it demonstrates high user engagement and an excellent safety profile.

PFM performance, measured by angle change and contraction hold time, improved over time. Established nomenclature defines PFMT as “exercises for improving PFM strength, endurance, power, and/or relaxation” [19]. It is possible that this digital PFMT program, guided by a motion-based device, addresses these multiple components, yielding effective outcomes. Furthermore, a greater angle change from baseline to follow-up was associated with clinically substantial symptom improvement. The contribution of PFM excursion to the restoration of the continence mechanism has been previously established [20,21]. The findings of this study suggest that the motion-based mechanism of this digital PFMT program may uniquely drive this component of pelvic floor rehabilitation.

Following software updates to improve the user experience, a greater proportion of users reported key demographic and clinical information [13]. Over 90% of users reported information about age, BMI, and race; 79.1% (749/947) and 67% (634/947) reported ethnicity and pregnancy history, respectively. Adherence was also improved in this study, with a greater proportion using the device 10 or more times per week. These improvements in data collection and user engagement followed key updates to the digital PFMT program informed by the evidence describing PFMT adherence and health behavior change, coupled with a user-centered design approach. A growing body of literature underscores the importance of leveraging clinical evidence and human-centered design in the creation of digital health tools [22-24]. These methodologies may continue to be applied to future program iterations to further optimize data capture and patient engagement. This evidence-informed approach can optimize real-world data collection and has the potential to transform population-level pelvic health research.

A key strength of this study includes the large sample that provided baseline and follow-up UI symptom data using multiple validated questionnaires, allowing for greater confidence in study outcomes. The product design enables passive data collection about usage and, thus, provides important information about PFMT performance in the real world. One limitation of this study is the lack of racial and ethnic diversity, which limits the generalizability of these findings. This may be improved through broader insurance coverage, enabling wider patient catchment. In addition, the significant association between adherence and favorable symptom improvement may be influenced by the large number of participants (n=880) in the ≥10 uses per week category compared with the lower adherence categories. Similarly, the low number of underweight (BMI <18.5 kg/m^2^) participants (n=9) may contribute to the lack of substantial findings in this group. Finally, the timing of UDI-6 follow-up varied, with most users reporting UDI-6 scores at 12 weeks. However, the variability in timing limits to conclusions about the optimal duration of use and symptom improvement. Future product development will optimize data collection, including both the completeness of key clinical information and consistent timing for the completion of outcome surveys. This will facilitate additional research in this context and may lend itself to predictive modeling to enhance patient selection and further improve UI treatment outcomes with this medical device and digital PFMT program.

### Conclusions

This study provides real-world evidence to support the effectiveness and safety of a prescribed digital health treatment program for UI using a motion-based device. First-line UI treatment, when implemented using this digital PFMT program, yields statistically and clinically significant symptom improvements across age and BMI categories and UI subtypes. Given the high prevalence of UI among women in the United States and the gross undertreatment of this condition, interventions that enable first-line treatment at scale are much needed. This represents one avenue to improve access to effective treatment and to address health disparities in UI care, particularly for those in geographic areas that lack specialized pelvic health clinicians or are otherwise unable to engage in in-person treatment due to employment, family, time, financial, or other constraints.

## Appendix

Multimedia Appendix 1 Additional analysis and results associated with adherence to the digital health program.

## Abbreviations

HIPAA: Health Insurance Portability and Accountability Act
*ICD-10*: *International Classification of Diseases, Tenth Revision*
MCID: minimum clinically important difference
PASS: Patient Acceptable Symptom State
PFM: pelvic floor muscle
PFMT: pelvic floor muscle training
PGI-I: Patient Global Impression of Improvement
UDI-6: Urogenital Distress Inventory-6 Short Form
UI: urinary incontinence

## Data Availability

The data sets generated during and/or analyzed during this study are available from the corresponding author upon reasonable request.
