# Correction: Real-World Evidence From a Digital Health Treatment Program for Female Urinary Incontinence: Observational Study of Outcomes Following User-Centered Product Design

**DOI:** 10.2196/65416

**Published:** 2024-08-27

**Authors:** Evelyn Hall, Laura Keyser, Jessica McKinney, Samantha Pulliam, Milena Weinstein

**Affiliations:** 1 Tufts University Medical Center Tufts University School of Medicine Boston, MA United States; 2 Department of Physical Therapy and Rehabilitation Science University of California, San Francisco San Francisco, CA United States; 3 Axena Health, Inc. Auburndale, MA United States; 4 College of Health and Human Services Andrews University Berrien Springs, MI United States; 5 Massachusetts General Hospital Harvard Medical School Boston, MA United States

In “Real-World Evidence From a Digital Health Treatment Program for Female Urinary Incontinence: Observational Study of Outcomes Following User-Centered Product Design” (JMIR Form Res 2024;8:e58551) the authors made one clarification and noted one error.

In the originally published article, the statistical assumptions applied in the adherence analysis were not fully defined.

A statement has been added to the second paragraph of Methods: Outcomes Analysis to clarify the assumptions applied in the original paper, and Supplement 1 has been added to provide an alternate analysis of adherence, as follows:

Original text:

Adherence to the prescribed PFMT regimen was averaged over a 12-week period and categorized into 3 groups, such as 0-4 uses per week, 5-9 uses per week, and ≥10 uses per week, with a maximum of 14 weekly uses.

Added statement:

Non-responses were considered missing data and excluded from analysis. Supplement 1 provides further analysis of adherence, in which non-responses were considered non-use (0 uses).

The authors also noted one error in reporting the proportion of users who experience vaginal irritation. This has been corrected in Results, as follows:

Original text:

Adverse events included vaginal irritation (3/1419, 2.3%), back pain (27/1419, 1.9%), yeast infection (10/1419, 0.7%), and urinary tract infection (9/1419, 0.6%).

Corrected text:

Adverse events included vaginal irritation (32/1419, 2.3%), back pain (27/1419, 1.9%), yeast infection (10/1419, 0.7%), and urinary tract infection (9/1419, 0.6%).

The correction will appear in the online version of the paper on the JMIR Publications website on August 27, together with the publication of this correction notice. Because this was made after submission to PubMed, PubMed Central, and other full-text repositories, the corrected article has also been resubmitted to those repositories.

## Appendix

Supplement 1 Additional analysis and results associated with adherence to the digital health program.

