# Supplementary material for: Real-World Evidence From a Digital Health Treatment Program for Female Urinary Incontinence: Observational Study of Outcomes Following User-Centered Product Design
Source: JMIR Form Res. 2024 Jun 27;8:e58551. doi: 10.2196/58551 (PMC11240060; doi:10.2196/58551)
Supplement: Multimedia Appendix 1 [file formative_v8i1e58551_app1.docx]

Correction: Real-World Evidence from a Digital Health Treatment Program for Female Urinary Incontinence: Observational Study of Outcomes Following User-Centered Product Design

**Supplement 1**

**Supplemental Methods**

In the originally published study, missing data was assumed missing at random. Non-responses were treated as missing data and excluded from adherence calculations. Mean adherence was calculated based on reported data. This supplement provides an additional analysis of adherence, in which missing data is assumed non-use (0 uses) and included in the adherence calculations. All adherence calculations were completed with this assumption applied, including mean and median adherence, proportion of users in each adherence category (0-4, 5-9, and ≥10 uses per week), and linear regression. A graphical representation of the mean weekly usage over 12 weeks was generated to depict the difference between these two analytic approaches.

**Supplemental Results**

Supplemental Table 1 summarizes median weekly adherence for users who provided follow-up urinary incontinence outcomes data and those who provided baseline data only. Those who provided outcomes data demonstrated significantly greater adherence than those who did not.

**Supplemental Table 1. Comparison of adherence for those who provided follow-up urinary incontinence outcomes data and those who provided baseline data only, missing adherence data assigned as non-use**

| **Demographic** | **Statistics** | **All participants** | **Baseline UDI-6 scores only** | **Baseline and follow-up UDI-6 scores** | ***P*-value*** |
| --- | --- | --- | --- | --- | --- |
|  |  | **n=1246** | **n=224** | **n=947** |  |
| Adherence  (Weekly uses over 12-week program, missing data attributed as non-use) | Median (IQR) | 6.3 (1.6-10.5) | 1.6 (0.6-2.5) | 7.8 (4.5-11.1) | **<.001** |

Supplemental Table 2 depicts change in UDI-6 scores from baseline to follow-up for the three adherence categories, 0-4, 5-9, and ≥10 uses per week, with a maximum of 14 weekly uses. Comparison across adherence categories demonstrates that all users improved. However, those in the 5-9 uses per week and ≥10 uses per week categories reported significantly greater UDI-6 score improvement (*P*<.001) and were more likely to reach the MCID compared with those in the 0-4 uses per week group.

| **Supplemental Table 2.** Urinary incontinence outcomes from a single cohort of real-world users: Urogenital Distress Inventory-6 Short Form (UDI-6) changes from baseline to follow-up by adherence category. ANOVA <.001, missing adherence data assigned as non-use | | | | | | |
| --- | --- | --- | --- | --- | --- | --- |
| **Adherence Category^** | **n** | **Baseline UDI-6** Mean ± SD | **UDI-6 at Last Follow-up** Mean ± SD | **Mean difference** ± SD | **p-value*** | **ANOVA** |
| 0-4 uses per week | 462 | 47.5 ± 19.4 | 39.2 ± 22.6 | -8.3 ± 19.1 | **<0.001** | **<0.001** |
| 5-9 uses per week | 325 | 46.3 ± 19.2 | 31.1 ± 20.0 | -15.2 ± 19.7 | **<0.001** |  |
| 10+ uses per week | 160 | 45.7 ± 19.3 | 33.7 ± 21.0 | -12.0 ± 21.0 | **<0.001** |  |
| UDI-6 = Urogenital Distress Inventory, Short Form; SD = Standard Deviation | | | | |  |  |
| * Paired t test  ^ Missing data attributed as non-use | | | | | | |

In multivariate logistic regression analysis, UI symptom severity as reported on the baseline UDI-6 and maximum angle change during PFM contraction were significantly associated with meeting the MCID. Age, BMI and UI subtype were not associated (Supplemental Table 3).

| **Supplemental Table 3.** Urinary incontinence outcomes from a single cohort of real-world users: factors associated with meeting the Urogenital Distress Inventory-6 Short Form (UDI-6) minimum clinically important difference; missing adherence data assigned as non-use | | | | |
| --- | --- | --- | --- | --- |
| **Covariates** | **Adjusted Odds Ratio** | **95% Confidence Limits** | | **p-value** |
| **Age** | 1.01 | 1.00 | 1.03 | 0.14 |
| **Body Mass Index** | 1.01 | 0.98 | 1.05 | 0.53 |
| **Baseline UDI-6 Score** | 1.04 | 1.03 | 1.0569 | **<0.001** |
| **Adherence (Weekly uses over 12-week program)** | 0.92 | 0.84 | 1.02 | 0.11 |
| **Baseline Maximum Angle Change with PFM Contraction** | 1.00 | 0.96 | 1.04 | 0.91 |
| **Follow-up Maximum Angle Change with PFM Contraction** | 1.04 | 1.01 | 1.06 | **<0.001** |
| **UI Subtype** |  |  |  |  |
| **Urgency UI** | Reference | - | - | - |
| **Mixed UI** | 0.77 | 0.33 | 1.78 | 0.54 |
| **Stress UI** | 1.16 | 0.53 | 2.52 | 0.71 |
| **Unspecified UI** | 0.62 | 0.27 | 1.43 | 0.26 |
| UDI-6 = Urogenital Distress Inventory, Short Form; PFM = Pelvic Floor Muscle; UI = Urinary Incontinence | | | | |

The mean weekly device usage over 12 weeks is presented in Supplemental Figures 1 and 2.

**Supplemental Figure 1. Mean weekly device usage over 12 weeks; missing data assigned as non-use; error bars equal one standard deviation**

**Supplemental Figure 2: Mean weekly device usage; missing data excluded; error bars equal one standard deviation**

Supplemental Figure 3 illustrates PFM angle change over time for those who did and did not meet the MCID.

**Supplemental Figure 3.** The maximum angle change with pelvic floor muscle (PFM) contraction depicted over time comparing those who did and did not meet the MCID on the UDI-6 remains significant. Individuals who met the MCID demonstrate greater angle change during PFM contraction compared with those who do not. This difference is evident early in their training and persists over time.
